# A Robust Current-Feedback Operational Amplifier-Based Front-End Amplifier for Electrocardiogram Signal Noise Removal

**DOI:** 10.3390/s26123665

**Published:** 2026-06-08

**Authors:** Suchada Sitjongsataporn, Panavy Pookaiyaudom, Phimchanok Sakunpongpitiporn, Pipat Sakarin, Panlop Puntuprecharat, Prajuab Pawarangkoon

**Affiliations:** 1 Department of Electronic Engineering, School of Electrical and Electronic Engineering (SEE), Mahanakorn University of Technology, Nongchok, Bangkok 10530, Thailand; ssuchada@mut.ac.th (S.S.); pipat.skr@gmail.com (P.S.); panlop@mut.ac.th (P.P.); prajuab@mut.ac.th (P.P.); 2 Center of Excellence in Sustainable Engineering, Mahanakorn University of Technology, Nongchok, Bangkok 10530, Thailand; phimchanok@mut.ac.th

**Keywords:** current-feedback operational amplifier (CFOA), electrocardiogram (ECG) signal, front-end amplifier, ECG noise removal

## Abstract

This paper introduces an electrocardiogram (ECG) noise removal front-end amplifier circuit based on a current-feedback operational amplifier (CFOA) that uses the current feedback to detect error signals and control the output. This ECG circuit focuses on denoising the ECG noise to accentuate the ECG electrical signals from the heart. Noises in ECG refer to baseline wander (BW), powerline interference (PLI) and motion artifacts. We proposed a CFOA-based ECG pre-amplifier using the AD844 commercial operational amplifier built inside with a positive second-generation current conveyor (CCII+) and a voltage follower circuit. This work introduces an ECG noise removal front-end amplifier based on a CFOA. The primary innovation lies in the balancing instrumentation amplifier architecture that utilizes the high-speed and robust properties of the AD844 commercial operational amplifier to achieve superior noise rejection. To protect against high-frequency interference, we introduce a novel cascaded low-pass filter (LPF) stage that ensures a sharper cut-off compared to traditional single-stage designs. Experimental results validate the design’s effectiveness, achieving a high common-mode rejection ratio (CMRR) of 75.4 dB and a mid-band gain of 46.5 dB. These performance metrics, combined with the circuit’s ability to eliminate BW and PLI, confirm its robust suitability for high-fidelity wearable ECG monitoring.

## 1. Introduction

Electrocardiogram (ECG) signal is a bioelectrical signal employed in cardiovascular monitoring from the human body [1] that is often corrupted by interference from respiratory or muscle movement and powerline interference. An ECG front-end amplifier is a kind of analog circuit design to detect the small and weak biopotential signals while removing ECG noise, such as powerline interference at 50/60 Hz, motion artifacts from respiration and muscle movement. In [2], a low-noise and low-power analog front-end (AFE) circuit was designed by a programmable gain amplifier to achieve the offset cancellation and a sigma-delta modulator to improve the stability for small biomedical signals. In [3], the authors have reviewed and discussed the multiple physiological signal acquisition circuits for wearable health monitoring devices. In [4], capacitive non-contact electrodes for bioelectrical electrocardiogram (ECG) signal monitoring and detection were proposed by using an AFE circuit. In [5], an automatic gain control has been modified with an AFE instrument amplifier for cardiac electrical signals to achieve high gain and high common-mode rejection ratio (CMRR).

Meanwhile, a low-power two-electrode recording system with a biopotential amplifier [6] was demonstrated for capturing small biomedical signals while enduring a large common-mode interference (CMI). In [7], the practical design of a biopotential AFE circuit for tolerating CMI was reviewed. In [8], a configurable current conveyor circuit was characterized for biomedical applications. MOS-based technology with the second generation of current conveyor (CCII) [9] was applied as a front-end double differential amplifier for superficial electromyography (EMG) application by placing electrode arrays on the skin against crosstalk noise signals. In [10], low-power and low-voltage techniques on CCII design were employed in biomedical applications. In [11], a CCII-based current buffer was designed for a biopotential read-out AFE circuit. In [12,13], a low-noise and low-power instrumentation amplifier was presented for a wearable ECG monitoring system.

For monitoring biomedical applications, a heart-rate system-on-chip for a long-term continuous cardiac monitoring system [14] was fabricated with low power consumption. In [15], a wireless ECG electrode with micropower consumption was embedded with Bluetooth Low Energy (BLE) connectivity. In [16], the capacitive ECG circuit was presented to recognize the electro-bio signal on clothing by a single lead heart rate-monitoring system with high impedance for capturing ECG signals.

From the works mentioned, recent developments in ECG front-end amplifiers have focused on balancing low power consumption with high signal integrity. However, each approach presents specific technical trade-offs as follows.

- Precision- and stability-oriented AFEs: Some designs utilize programmable gain amplifiers (PGAs) and sigma-delta modulators to achieve high stability and offset cancellation. While these provide excellent resolution for small signals, they often require complex digital post-processing and higher circuit complexity to maintain stability.
- Interference-tolerant instrumentation amplifiers: Approaches using automatic gain control (AGC) or specialized two-electrode recording systems were developed to achieve high CMRR and tolerate large common-mode interference (CMI). Although effective at rejecting baseline noise, these voltage-mode instrumentation amplifiers often face limitations in bandwidth and gain-bandwidth product (GBP).
- Current-mode architectures: The use of second-generation current conveyors (CCII) has gained traction due to their high-speed performance and low-voltage operation. However, many existing CCII-based designs require complex mathematical derivations for second-order filtering stages, which can complicate the design and tuning process for specific noise environments like high-frequency muscle artifacts.

To address these limitations, this paper proposes a front-end amplifier based on the current-feedback operational amplifier (CFOA), which combines the high-speed benefits of current-mode processing with the simplicity of voltage-mode feedback. The key improvements of this design include:

1. Simplified band-pass architecture: Unlike conventional designs that rely on complex second-order filtering calculations, this work strategically decomposes the bandpass function into simplified first-order high-pass and low-pass stages. This allows for easier parameter adjustment while maintaining high performance.
2. Enhanced muscle artifact rejection: While many wearable designs focus only on powerline interference, our architecture introduces a cascaded LPF stage. This provides a significantly sharper high-frequency cut-off (targeting the 150–200 Hz range) to more effectively eliminate muscle artifacts that frequently corrupt wearable ECG data.
3. Robust balancing configuration: By implementing a balancing instrumentation amplifier structure using the AD844 commercial operational amplifier, the design achieves a superior CMRR of 75.4 dB and a mid-band gain of 46.5 dB, ensuring high-fidelity signal recovery in noisy environments.

**Figure 1:**
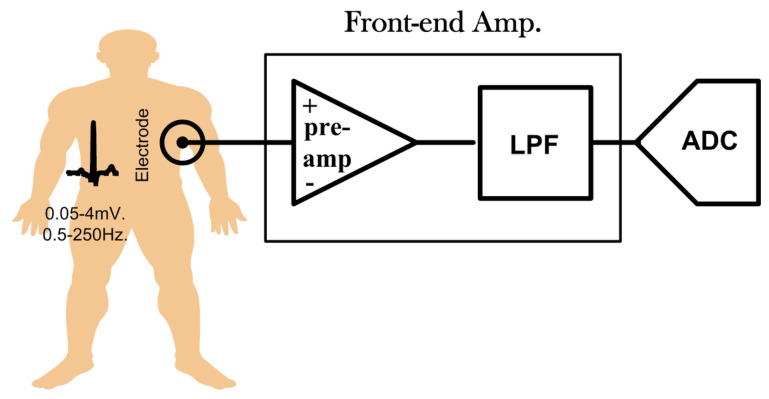
ECG measurement.

This paper focuses on how to design an ECG front-end amplifier, as shown in Figure 1. The proposed ECG front-end low-noise instrumentation amplifier consists of an ECG pre-amplifier and a low-pass filter (LPF) for removing the corrupted ECG signals.

## 2. Current-Feedback Operational Amplifier Architecture

**Figure 2:**
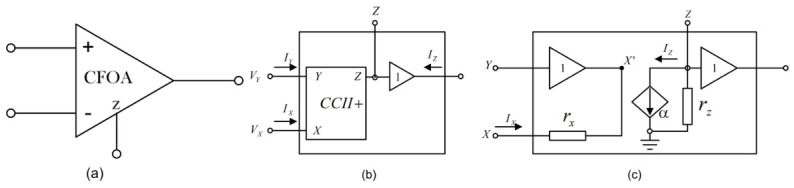
CFOA based on AD844 commercial operational amplifier: (**a**) symbol; (**b**) small signal equivalent circuit and (**c**) non-ideal equivalent circuit affected by $R_{x}$ and $R_{y}$.

The current-feedback operational amplifier [17] is a kind of electronic amplifier that uses current feedback to detect error signals and control the output. The block diagram of a CFOA based on an AD844 commercial operational amplifier is shown in Figure 2. A small signal equivalent circuit of CFOA consists of a positive second-generation current conveyor (CCII+) connected with a voltage follower, as shown in Figure 2b. An input voltage $v_{y}$ is connected to a voltage follower, which produces a current *i* through a resistor $r_{x}$.

The CCII+ is expressed in the matrix form [18] as

(1) $$\left[\begin{array}{c} I_{y}\\ V_{x}\\ I_{z} \end{array}\right]=\left[\begin{array}{ccc} 0 & 0 & 0\\ 1 & 0 & 0\\ 0 & \alpha & 0 \end{array}\right]\cdot \left[\begin{array}{c} V_{y}\\ I_{x}\\ V_{z} \end{array}\right]$$

that an ideal CCII+ is under condition as: $r_{x}=0,r_{z}=\infty$, then $i_{x}=i_{z}$ and α=1 as shown in Figure 2b. A non-ideal CCII small-signal model in Figure 2c includes these parasitics to predict performance degradation in amplifiers, where $r_{y}$ is ideally infinite at input terminal-Y and $r_{x}$ is a small value non-zero resistance at input terminal-X, typically 10–100 Ω.

## 3. Proposed CFOA-Based ECG Pre-Amplifier

**Figure 3:**
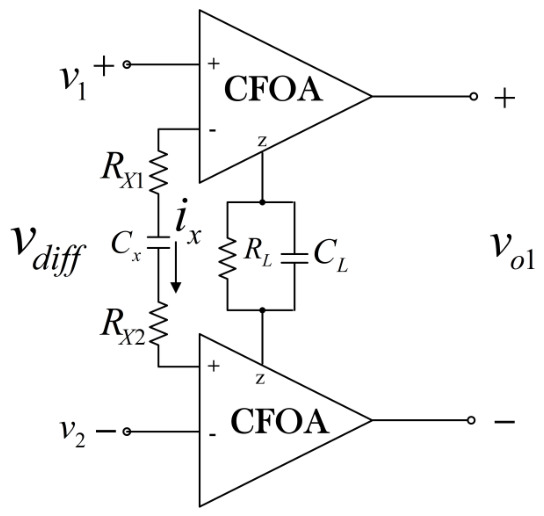
Proposed CFOA-based ECG pre-amplifier for noise removal.

In this section, the proposed CFOA-based ECG bandpass pre-amplifier is designed in the form of a balancing instrumentation amplifier for noise removal as shown in Figure 3. The use of a CFOA architecture, such as the AD844, offers fundamental performance advantages over traditional voltage-feedback amplifiers for biopotential acquisition. Unlike voltage-mode amplifiers, which are constrained by a constant gain-bandwidth product, the CFOA’s bandwidth is independent of the closed-loop gain. This decoupling allows the front end to maintain a stable frequency response even at the high gain (46.5 dB) required for ECG signals. Additionally, the CFOA’s high slew rate and current-sensing input stage provide superior dynamic range and common-mode rejection, which are essential for preserving the rapid transitions of the QRS complex while suppressing environmental interference.

As shown in Figure 3, the input current $i_{x}$ is calculated as follows.

(2) $$\begin{array}{c} i_{x}=\frac{v_{1}-v_{2}}{R_{x_{1}}+Z_{C_{x}}+R_{x_{2}}}=\frac{v_{diff}}{2R_{x}+\frac{1}{sC_{x}}} \end{array}$$

where an impedance $Z_{C_{x}}=\frac{1}{sC_{x}}\Omega$ and s=jω rad/s. We assume that $R_{x}=R_{x_{1}}=R_{x_{2}}$, where $r_{x}$ is a low resistance at input stage.

Based on CCII+ properties, a current $i_{z}$ is equal to $i_{x}$. So, a current $i_{z}$ can be written as

(3) $$\begin{array}{c} i_{z}=i_{x}=v_{diff}\cdot \frac{sC_{x}}{\left(2sC_{x}R_{x}+1\right)} \end{array}$$

At the output part in Figure 3, an output voltage $v_{o_{1}}$ can be calculated by

(4) $$\begin{array}{c} v_{o_{1}}=i_{z}\cdot \frac{R_{L}Z_{C_{L}}}{R_{L}+Z_{C_{L}}} \end{array}$$

where an impedance $Z_{C_{L}}=\frac{1}{sC_{L}}\Omega$.

By substituting $Z_{C_{L}}$ into (3), we have

(5) $$\begin{array}{c} v_{o_{1}}=i_{z}\cdot \frac{R_{L}/sC_{L}}{R_{L}+\frac{1}{sC_{L}}}=i_{z}\cdot \frac{R_{L}}{\left(sC_{L}R_{L}+1\right)} \end{array}$$

Replacing (3) with (5), we get

(6) $$\begin{array}{c} v_{o_{1}}=v_{diff}\cdot \frac{sC_{x}}{\left(2sC_{x}Rx+1\right)}\cdot \frac{R_{L}}{\left(sC_{L}R_{L}+1\right)} \end{array}$$

From (6), we can find a transfer function T(s) of proposed CFOA-based ECG band-pass pre-amplifier as

(7) $$\begin{array}{c} T\left(s\right)=\frac{v_{o_{1}}}{v_{diff}}=\frac{sC_{x}}{\left(2sC_{x}R_{x}+1\right)}\cdot \frac{R_{L}}{sC_{L}R_{L}+1} \end{array}$$

According to the second order filtering [19], a band-pass filter (BPF) function is usually obtained in the standard form as

(8) $$\begin{array}{c} T_{s}\left(s\right)=\frac{a_{1}\cdot s}{s^{2}+s\frac{\omega_{0}}{Q}+\omega_{0}^{2}} \end{array}$$

where a center-frequency gain $H_{s_{0}}$ of BPF is

(9) $$\begin{array}{c} H_{s_{0}}=\frac{a_{1}\cdot Q}{\omega_{0}} \end{array}$$

Thus, we can reorganize (7) as standard in (8) as

(10) $$\begin{array}{cc} \tilde{T}\left(s\right) & =\frac{sC_{x}R_{L}}{2s^{2}R_{x}C_{x}R_{L}C_{L}+s\left(2R_{x}C_{x}+R_{L}C_{L}\right)+1}\\ & =\frac{s}{2R_{x}C_{L}\left(s^{2}+s\frac{\left(2R_{x}C_{x}+R_{L}C_{L}\right)}{2R_{x}C_{x}R_{L}C_{L}}+\frac{1}{2R_{x}C_{x}R_{L}C_{L}}\right)} \end{array}$$

where

(11) $$\begin{array}{cc} a_{1} & =\frac{1}{2R_{x}C_{L}} \end{array}$$

(12) $$\begin{array}{cc} \frac{\omega_{0}}{Q} & =\frac{\left(2R_{x}C_{x}+R_{L}C_{L}\right)}{\left(2R_{x}C_{x}R_{L}C_{L}\right)} \end{array}$$

Then, we can find ${\tilde{H}}_{0}$ of BPF by replacing (11) and (12) with (9) as

(13) $$\begin{array}{c} {\tilde{H}}_{0}=\frac{1}{2R_{x}C_{L}}\cdot \frac{2R_{x}C_{x}R_{L}C_{L}}{\left(2R_{x}C_{x}+R_{L}C_{L}\right)}=\frac{C_{x}R_{L}}{2R_{x}C_{x}+R_{L}C_{L}} \end{array}$$

**Assumption** **1.** *We assume that a load capacitor $C_{L}$ is much less than a capacitor $C_{x}$ at port x.*

Therefore, a gain ${\tilde{H}}_{0}$ of BPF can be expressed by following Assumption 1 as

(14) $$\begin{array}{c} {\tilde{H}}_{0}≃\frac{R_{L}}{2R_{x}} \end{array}$$

where $C_{L}\ll C_{x}$.

As seen above, we avoid the complex calculation to find a low frequency cut-off $\omega_{C_{L}}$ and a high frequency cut-off $\omega_{C_{H}}$ from the second order band-pass filtering function by using the first-order high-pass filter (HPF) and low-pass filter (LPF).

According to the first-order filtering function [19], HPF and LPF are expressed in the standard form as

(15) $$\begin{array}{cc} T_{HPF}\left(s\right) & =\frac{{\tilde{a}}_{1}\cdot s}{s+\omega_{C_{L}}} \end{array}$$

(16) $$\begin{array}{cc} T_{LPF}\left(s\right) & =\frac{{\tilde{a}}_{0}}{s+\omega_{C_{H}}} \end{array}$$

From (7), we assume that a transfer function T(s) of the proposed band-pass amplifier can be created with the characteristics of the HPF with $\omega_{C_{L}}$ connected to the LPF with $\omega_{C_{H}}$ as

(17) $$\begin{array}{cc} T(s) & =\underset{\mathrm{HPF}}{\underset{︸}{\frac{sC_{x}}{\left(2sC_{x}R_{x}+1\right)}}}\cdot \underset{\mathrm{LPF}}{\underset{︸}{\frac{R_{L}}{sC_{L}R_{L}+1}}} \end{array}$$

So, we can explore the transfer functions of HPF and LPF as

(18) $$\begin{array}{cc} {\tilde{T}}_{HPF}\left(s\right) & =\frac{sC_{x}}{2sC_{x}R_{x}+1} \end{array}$$

(19) $$\begin{array}{cc} {\tilde{T}}_{LPF}\left(s\right) & =\frac{R_{L}}{sC_{L}R_{L}+1} \end{array}$$

By comparing (18) in the form of (15), ${\tilde{T}}_{HPF}\left(s\right)$ is rewritten as

(20) $$\begin{array}{c} ∴{\tilde{T}}_{HPF}\left(s\right)=\frac{sC_{x}}{2R_{x}C_{x}\left(s+\frac{1}{2R_{x}C_{x}}\right)}=\frac{s}{2R_{x}\left(s+\frac{1}{2R_{x}C_{x}}\right)} \end{array}$$

where

(21) $$\begin{array}{c} {\tilde{\omega }}_{C_{L}}=\frac{1}{2R_{x}C_{x}} \end{array}$$

Therefore, the low frequency cut-off ${\tilde{f}}_{C_{L}}$ can be expressed by following (21) as

(22) $$\begin{array}{c} ∴{\tilde{f}}_{C_{L}}=\frac{1}{4\pi R_{x}C_{x}} \end{array}$$

In a similar way, the high frequency cut-off ${\tilde{f}}_{C_{H}}$ from (19) can be obtained by following (16) as

(23) $$\begin{array}{cc} ∴{\tilde{T}}_{LPF}\left(s\right) & =\frac{R_{L}}{R_{L}C_{L}\left(s+\frac{1}{R_{L}C_{L}}\right)} \end{array}$$

where

(24) $$\begin{array}{cc} {\tilde{\omega }}_{C_{H}} & =\frac{1}{R_{L}C_{L}} \end{array}$$

Therefore, the high frequency cut-off ${\tilde{f}}_{C_{H}}$ can be expressed by following (24) as

(25) $$\begin{array}{cc} ∴{\tilde{f}}_{C_{H}} & =\frac{1}{2\pi R_{L}C_{L}} \end{array}$$

While the general form of the transfer function in (8)–(13) allows for various filtering characteristics, the component values in this design are strategically selected to avoid complex-conjugate poles. By ensuring that the poles are widely separated and real, the system achieves a wide-band frequency response suitable for diagnostic ECG acquisition. This behavior is more precisely detailed in (15)–(25) and is empirically validated by the AC analysis, which shows a flat pass-band free from high-Q resonance peaks.

## 4. Proposed CFOA-Based Front-End ECG Amplifier

**Figure 4:**
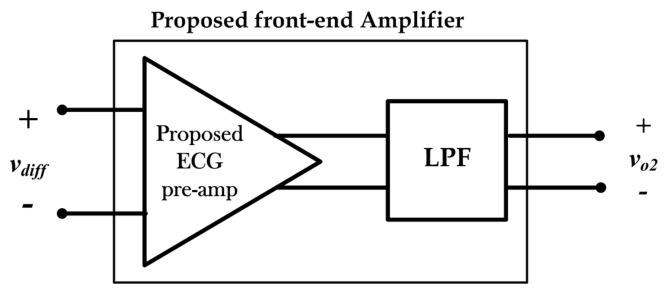
Concept of proposed CFOA-based front-end ECG amplifier.

According to [20,21], motion artifact and ECG noise occupy the frequency range from below 1 Hz to 150 Hz. For example, the ECG high frequency range is coming from a muscle artifact, which is typically set at 150–200 Hz. To protect the high frequency noise, we introduce the cascaded low-pass filter (LPF) to make a sharper high frequency cut-off. In order to take the sharp cut-off high-frequency, we introduce the concept of the proposed CFOA-based front-end ECG amplifier based on the proposed CFOA-based ECG pre-amplifier cascaded with LPF, as shown in Figure 4.

**Figure 5:**
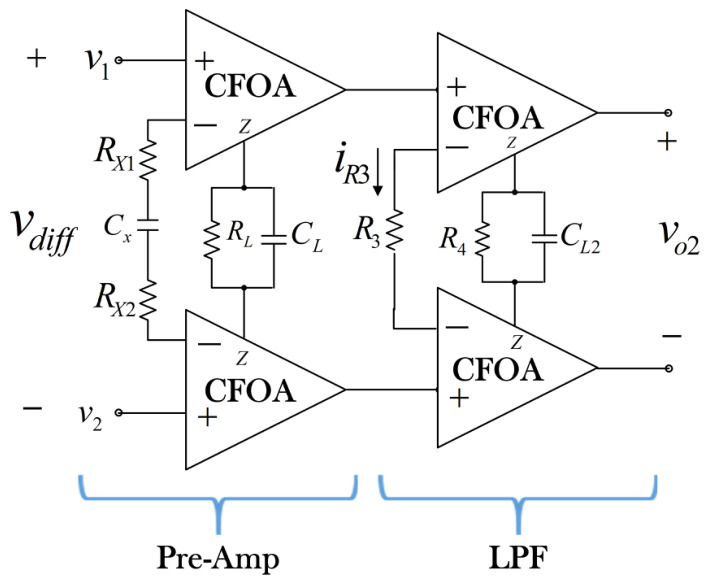
Proposed CFOA-based front-end ECG amplifier by adding LPF.

In this section, we propose a CFOA-based front-end ECG amplifier by extending the ECG pre-amplifier cascaded with the LPFk, as shown in Figure 5. At LPF, a current $i_{R_{3}}$ and $v_{o}$ can be calculated by

(26) $$\begin{array}{cc} i_{R_{3}} & =\frac{v_{R_{3}}}{R_{3}} \end{array}$$

(27) $$\begin{array}{cc} v_{o_{2}} & =i_{R_{3}}\cdot \frac{R_{4}\cdot Z_{C_{L_{2}}}}{R_{L}+Z_{C_{L_{2}}}}=\frac{v_{R_{3}}}{R_{3}}\cdot \frac{R_{4}/sC_{L_{2}}}{R_{4}+1/sC_{L_{2}}} \end{array}$$

where $Z_{C_{L_{2}}}=1/sC_{L_{2}}\Omega$.

By substituting (26) into (27), we arrive at

(28) $$\begin{array}{cc} v_{o_{2}} & =\frac{v_{R_{3}}}{R_{3}}\cdot \frac{R_{4}/sC_{L_{2}}}{R_{4}+1/sC_{L_{2}}}\\ \; & =v_{R_{3}}\cdot \frac{R_{4}}{R_{3}\left(sR_{4}C_{L_{2}}+1\right)} \end{array}$$

From (28), a transfer function of LPF $T_{LPF}$ can be expressed by

(29) $$\begin{array}{c} ∴T_{LPF}=\frac{v_{o_{2}}}{v_{R_{3}}}=\frac{R_{4}}{R_{3}\left(sR_{4}\;C_{L_{2}}+1\right)} \end{array}$$

According to the first-order LPF in (16), we reorganize (28) in term of (16) as

(30) $$\begin{array}{cc} T_{LPF} & =\frac{v_{o_{2}}}{v_{R_{3}}}=\frac{1/R_{x}\;C_{L_{2}}}{\left(s+1/R_{4}\;C_{L_{2}}\right)} \end{array}$$

(31) $$\begin{array}{cc} {\hat{\omega }}_{C_{H}} & =\frac{1}{R_{4}\;C_{L_{2}}} \end{array}$$

Similarly, the high frequency cut-off ${\hat{f}}_{C_{H}}$ can be obtained by following (31) as

(32) $$\begin{array}{c} ∴{\hat{f}}_{C_{H}}=\frac{1}{2\pi R_{4}\;C_{L_{2}}} \end{array}$$

where a gain of LPF ${\hat{a}}_{0}$ is given by

(33) $$\begin{array}{c} {\hat{a}}_{0}=\frac{R_{4}}{R_{3}} \end{array}$$

The optimal values are selected to match the standard diagnostic ECG bandwidth (approx. 0.5 Hz to 150 Hz).

- For $f_{L}\approx 0.8$ Hz: using $R_{x}$ = 1 kΩ and $C_{x}$ = 100 μF targets the suppression of the baseline wandering noise typically found below 1 Hz.
- For $f_{H}\approx$ 190 Hz: the combination of the pre-amp load ($R_{L}$ = 500 kΩ, $C_{L}$ = 100 nF and the LPF ($R_{4}$ = 3 kΩ, $C_{L_{2}}$ = 200 nF) is optimized to provide a sharp roll-off starting near 150–200 Hz to eliminate muscle artifacts.
- For Gain ≅46.5 dB: the resistance ratios (specifically the high $\frac{R_{L}}{2\;R_{x}}$ ratio) ensure that the weak biopotential signal (0.05–4 mV) is amplified sufficiently for analog-to-digital converter (ADC) processing while maintaining a high signal-to-noise ratio (SNR).

While CFOAs are typically recognized for high-bandwidth applications, they are utilized in this low-frequency ECG acquisition system due to their architectural simplicity in implementing frequency-selective stages. Specifically, the CFOA structure enables the easy setting of the upper cut-off frequency through simple RC combinations at the high-impedance compensation nodes ($R_{L}+C_{L}$ and $R_{4}+C_{L_{2}}$). This approach provides a robust and predictable frequency response while maintaining a high slew rate, which is critical for preserving the rapid transitions of the QRS complex.

## 5. Characteristics of ECG Noise

**Figure 6:**
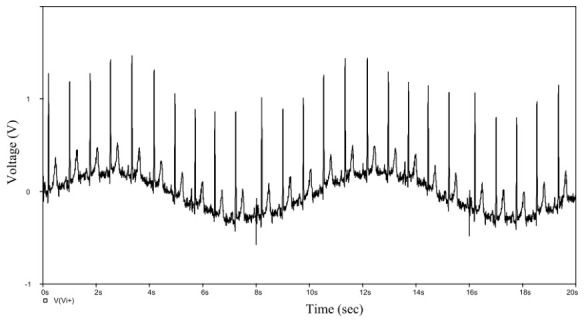
ECG contaminated with baseline wander at <1 Hz by PSPICE.

**Figure 7:**
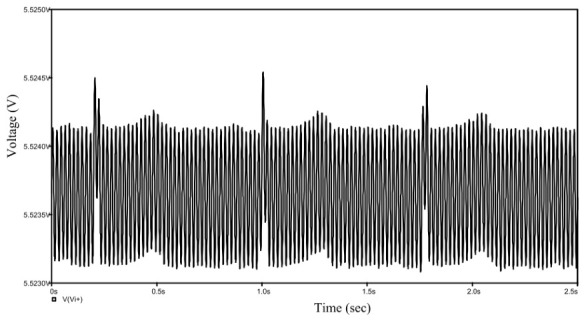
ECG corrupted by standard powerline frequency at 50 Hz by PSPICE.

**Figure 8:**
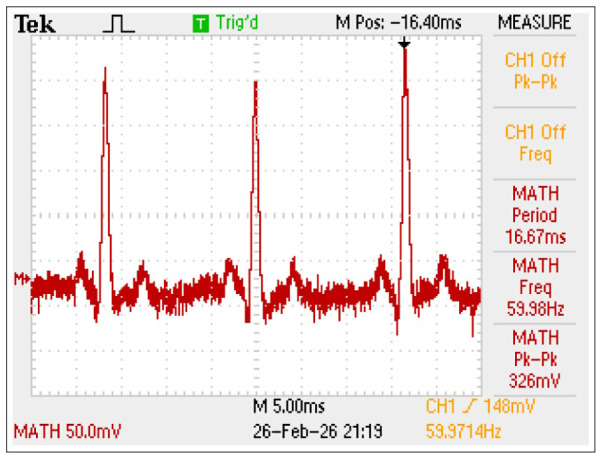
Electrode–skin impedance imbalance effect.

ECG signal is often corrupted by interference from respiratory or muscle movement and powerline interference. ECG noise signal refers to electrode–skin contact impedance, baseline wander (BW), powerline interference (PLI) and motion artifacts. Figure 6 shows the ECG contaminated with baseline wander at <1 Hz. It is noticed that the contamination of an ECG signal at <1 Hz is often caused by breathing. Figure 7 shows the ECG corrupted with powerline interference at 50 Hz simulated by PSPICE 9.1 simulation software. Figure 8 presents that the electrode–skin imbalance happens when the electrical contact quality differs between electrodes, causing significant noise and potential errors in biomedical recordings.

To validate the robustness of the proposed circuit, a comprehensive test signal was constructed by superimposing the individual disturbances described in Figure 6, Figure 7 and Figure 8 onto a standard clean ECG waveform. This composite signal, which includes 0.5 Hz baseline fluctuations, 50 Hz powerline noise, and high-frequency muscle artifacts, was used as the input for the performance demonstrations shown in the following sections. This ensures that the results reflect the circuit’s performance in a realistic, multi-noise environment.

It should be noted that while the differential frequency response remains flat around 50 Hz to preserve cardiac signal integrity, the 50 Hz powerline interference is effectively eliminated by the architecture’s high CMRR. By treating the AC network influence as a common-mode signal, the balancing structure provides 75.4 dB of rejection, precluding the need for a 50 Hz notch filter and avoiding the associated phase non-linearity.

## 6. Simulation Results

Circuit design is composed of a pre-amplifier stage and an LPF stage, shown in Figure 5, where the corresponding component values are shown in Table 1.

### 6.1. Results of Proposed CFOA-Based ECG Pre-Amplifier

**Figure 9:**
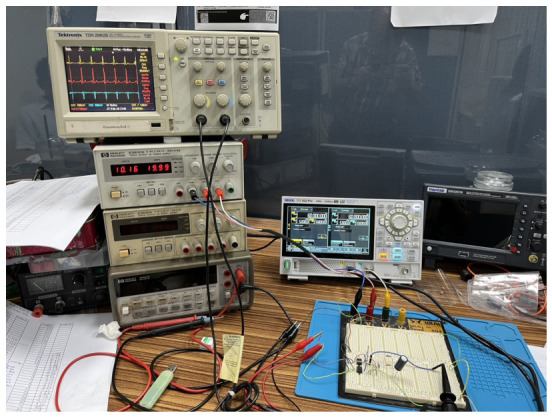
Setup environment for ECG measurement.

For simulation, we proposed a CFOA-based ECG pre-amplifier using an AD844 operational amplifier built inside with CCII+ and a voltage follower circuit. PSPICE software was used and the differential mode input voltage was 5 mV. For the practical experiment, we used the function/arbitrary waveform generators [22] that include pre-programmed ECG corrupted signals. Our setup environment for ECG measurement is shown in Figure 9.

**Figure 10:**
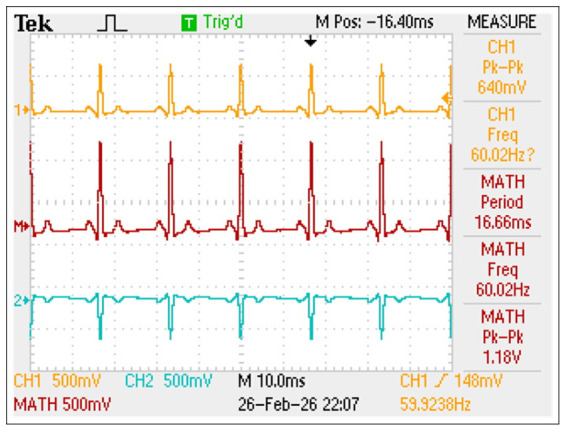
ECG output through proposed ECG pre-amplifier.

The experimental results confirm that the proposed circuit can effectively reconstruct the ECG waveform. The output in Figure 10 is deemed satisfactory, as it preserves the critical timing and amplitude features of the QRS complex, while achieving a clean, horizontal baseline.

### 6.2. Results of Proposed CFOA-Based Front-End ECG Amplifier

**Figure 11:**
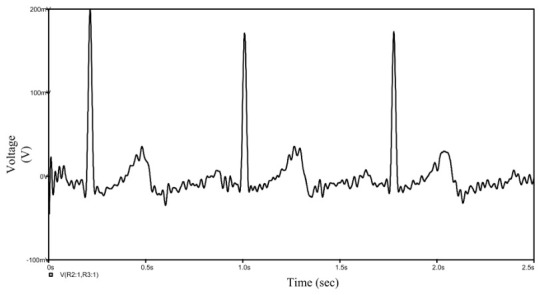
ECG output signal through proposed front-end ECG amplifier circuit in PSPICE.

For PSPICE simulation, we proposed a CFOA-based front-end ECG amplifier using an AD844 operational amplifier built inside with CCII+ and a voltage follower circuit. Figure 11 shows the ECG output signal passed through the proposed CFOA-based front-end ECG amplifier. The simulation result of the proposed system was conducted in two stages. Initially, the raw ECG signal, corrupted by baseline wander and 50 Hz interference, was processed by the CFOA-based pre-amplifier. This stage successfully recovered the ECG morphology and provided a mid-band gain of 46.5 dB. To achieve a high-fidelity diagnostic trace, this intermediate signal was then passed through the cascaded low-pass filter to suppress high-frequency muscle artifacts. Figure 11 illustrates the final output of the complete front-end system. As shown, the combined architecture effectively eliminates both low-frequency baseline fluctuations and high-frequency noise, resulting in a smooth and stable ECG waveform suitable for clinical interpretation.

In a fully differential circuit, the common mode rejection ratio (CMRR) is used to quantify the circuit’s design ability to reject common-mode interference signals. It is defined as the ratio of differential-mode gain ($A_{dm}$) to common-mode gain ($A_{cm}$), typically in units of decibels (dB) as

(34) $$\begin{array}{c} CMRR\left(dB\right)=A_{dm}\left(dB\right)-A_{cm}\left(dB\right) \end{array}$$

**Figure 12:**
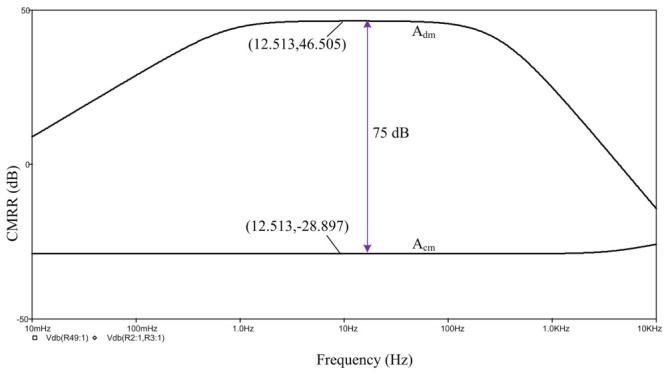
Common mode rejection ratio (CMRR) in dB of proposed CFOA-based front-end ECG amplifier.

Figure 12 represents simulation results obtained using PSPICE software. The following specific conditions and inputs were used to record these frequency characteristics:

- Differential mode input ($v_{diff}$): a voltage of 5 mV was applied. This input is used to determine the differential-mode gain ($A_{dm}$), which was recorded at 46.505 dB.
- Common mode input: while the specific common-mode input voltage is not explicitly listed as a single value, the resulting common-mode gain ($A_{cm}$) was measured at −28.897 dB to calculate the overall CMRR.
- Operating environment: the simulation utilized the AD844 commercial operational amplifier model, which incorporates an internal positive second-generation current conveyor (CCII+) and a voltage follower.
- Component values: the simulation followed the specific values listed in Table 1 (e.g., $R_{x}$ = 1 kΩ, $R_{L}$ = 500 kΩ, and $C_{x}$ = 100 μF).

These conditions resulted in a total CMRR of 75.4 dB.

**Figure 13:**
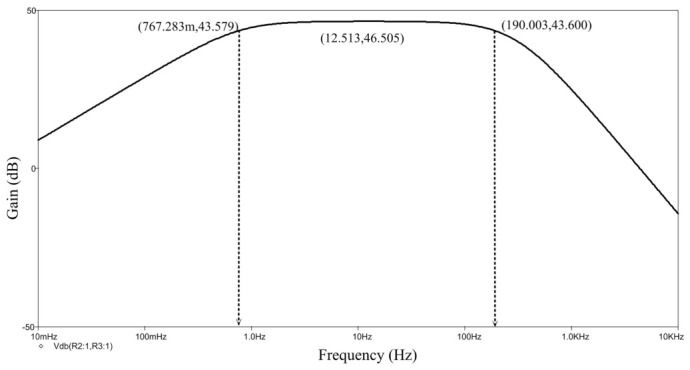
Frequency response of proposed CFOA-based front-end ECG amplifier.

Figure 13 presents the frequency response of the proposed front-end ECG amplifier with the mid-band gain at 46.5 dB. It is seen that the range of bandwidth is 0.767–190 Hz.

**Figure 14:**
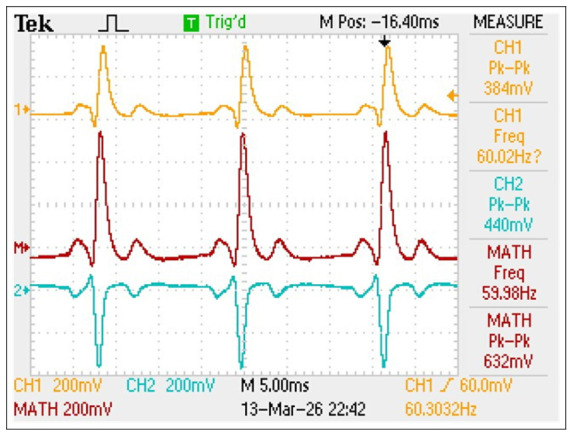
Experimental results showing the comparison between the corrupted input signal and the final processed ECG output, demonstrating successful noise removal and morphology preservation.

As shown in Figure 14, the front end successfully extracts the ECG signal from the noise. The output trace confirms that the P-QRS-T complexes are clearly defined and free from baseline fluctuations.

**Figure 15:**
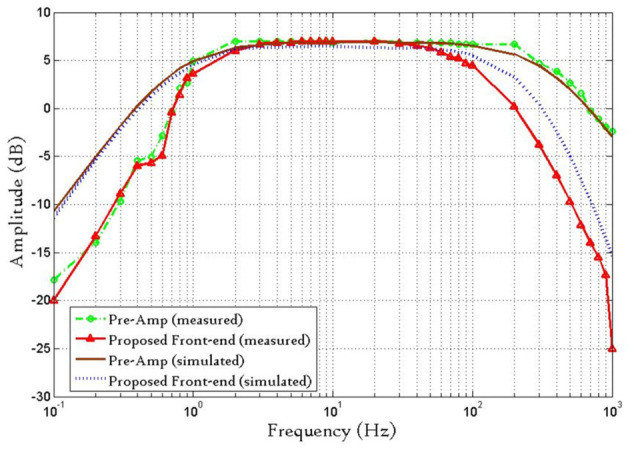
Frequency responses of proposed ECG pre-amplifier (pre-amp) and proposed front-end ECG amplifier (proposed front-end).

Figure 15 shows the comparison of simulated and measured frequency responses of the proposed CFOA-based ECG pre-amplifier and the proposed CFOA-based front-end ECG amplifier. The proposed front-end ECG amplifier is designed with an ideal mid-band gain of 7 dB and a bandwidth of 350 Hz. Both the simulation and experimental results show a relatively flat mid-band response close to the designed gain, indicating that the circuit operates as intended. When the LPF is cascaded, the overall bandwidth becomes narrower, while maintaining approximately the same mid-band gain. The added LPF is designed with a DC gain of 0 dB and a cut-off frequency of 250 Hz. Both simulated and measured results clearly show the expected attenuation beyond the cut-off frequency.

To evaluate the effectiveness of the proposed CFOA-based front-end amplifier, its overall performance is summarized and compared with recently reported ECG acquisition systems in Table 2. The comparison focuses on key metrics such as voltage gain, bandwidth, and CMRR, which are critical for high-fidelity biopotential recording.

As demonstrated in Table 2, this work achieves a superior mid-band gain of 46.5 dB and a high CMRR of 75.4 dB, significantly improving the signal-to-noise ratio in the presence of heavy environmental interference. Furthermore, the selection of a 190 Hz upper cut-off frequency ensures that the diagnostic integrity of the QRS complex is maintained while effectively suppressing high-frequency muscle artifacts. Despite these advantages, there are clear areas for improvement. While the current discrete prototype using the AD844 successfully validates the robustness of the balancing architecture and the simplified filtering strategy, the total power consumption remains higher than that of fully integrated solutions. Therefore, future work will focus on migrating this topology from discrete components to a dedicated low-power CMOS integrated circuit (IC). By implementing the CFOA structure at the transistor level using nanopower design techniques, the power-to-performance ratio can be optimized, making the system more suitable for long-term, battery-operated wearable monitoring applications.

The upper 3 dB cut-off frequency of the proposed front end is strategically set at 190 Hz. This selection represents a deliberate design trade-off between signal fidelity and noise immunity. While the diagnostic features of the ECG signal are primarily concentrated below 150 Hz, high-frequency muscle artifacts often emerge in the 150–200 Hz range. By positioning the cut-off at 190 Hz, the circuit preserves the sharp transitions of the QRS complex while providing significant attenuation for high-frequency interference, as evidenced by the frequency characteristics shown in Figure 13 and Figure 15.

## 7. Discussion

It is noted that some deviations between simulation and measurement can be observed at the low- and high-frequency regions. At high frequencies, the measured response rolls off earlier than the simulated response due to parasitic resistances at the X-terminal of the AD844. Meanwhile, the discrepancies at very low frequencies are mainly caused by the parasitic resistance at the Y-terminal of AD844, which slightly affects the input impedance and low-frequency gain. Despite these non-ideal effects, the experimental results generally agree well with the simulation, confirming the correct operation of the proposed circuit.

In the modern era of biomedical instrumentation, there is a recurring debate regarding the necessity of complex analog front ends versus digital signal processing (DSP). While DSP is capable of sophisticated filtering, the analog stage serves as the gatekeeper of signal integrity. Without high-precision analog rejection of common-mode noise and baseline wander, the signal’s information can be lost during digitization due to ADC saturation. Furthermore, while dedicated ECG ICs offer compact solutions, the proposed CFOA-based architecture provides a high degree of design flexibility and transparency. By leveraging discrete current-feedback topologies, designers can achieve superior CMRR and customizable frequency shaping that is often restricted in fixed-architecture commercial chips, making this approach highly suitable for specialized research and low-power sensing applications.

While the current results are based on experimental validation using calibrated ECG sources and real-world noise models, they provide a necessary baseline for the circuit’s performance. The use of a controlled input environment allows for a precise evaluation of the CFOA architecture’s CMRR and filtering accuracy without the stochastic interference of human-subject variability. Future work will focus on clinical validation and in vivo testing to assess the system’s performance across diverse skin–electrode interface conditions.

## 8. Conclusions

This paper proposes a CFOA-based front-end ECG amplifier for ECG noise removal. Circuit design was derived in terms of small-signal analysis. The characteristics of ECG noises are described in the simulation. The proposed circuit was tested and validated by the PSPISE simulation software. Both experimental results in the practical simulation and measurement show that the proposed CFOA-based front-end ECG amplifier has a high CMRR of 75 dB and a mid-band gain of 47.5 dB. It is confirmed that the proposed circuit is suitable for a wearable device.

While many state-of-the-art ECG front ends rely on complex voltage-mode instrumentation or multi-stage current-mode topologies, this work demonstrates that a simplified CFOA-balancing architecture can meet and exceed diagnostic requirements. By achieving a 46.5 dB mid-band gain and a 75.4 dB CMRR, the proposed circuit provides a robust solution for noise-heavy environments. The key contribution is the ability to maintain high signal integrity and precise frequency shaping through an efficient, low-complexity design that simplifies the transition from theory to physical implementation.

## Figures and Tables

**Table 1:** Component values.

Item	Value	Item	Value
$R_{x_{1}},R_{x_{2}}$	1 kΩ	$R_{3},R_{4}$	3 kΩ
$R_{L}$	500 kΩ	$C_{L_{2}}$	200 nF
$C_{x},C_{L}$	100 μF, 100 nF		

**Table 2:** Performance comparison of the proposed ECG front-end amplifier with recent works.

Parameter	Ref. [11]	Ref. [18]	Ref. [20]	This Work
Technology	CMOS	CCII-based	Wearable Flexible	CFOA (AD844)
Gain (dB)	40	34.5	40	46.5
Bandwidth (Hz)	0.5–200	0.5–150	0.05–100	0.767–190
CMRR (dB)	>60	68	N/A	75.4
Architecture	Voltage-mode	Current-mode	Hybrid	Current-feedback
Simplicity	High	Medium	High	Very High

## Data Availability

The original contributions presented in this study are included in the article. Further inquiries can be directed to the corresponding author.

## Abbreviations

The following abbreviations are used in this manuscript:

AFE	Analog front end
BPF	Band-pass filter
BW	Baseline wander
CCII	Second-generation current conveyor
CCII+	Positive second-generation current conveyor
CFOA	Current Feedback Operational Amplifier
CMI	Common-mode interference
CMRR	Common Mode Rejection Ratio
ECG	Electrocardiogram
EMG	Electromyography
HPF	High-pass filter
LPF	Low-pass filter
PLI	Powerline interference

## References

[1] Martinek R., Ladrova M., Sidikova M., Jaros R., Behbehani K., Kahankova R., Kawala-Sterniuk A. (2021). Advanced Bioelectrical Signal Processing Methods: Past, Present and Future Approach-Part I: Cardiac Signals. Sensors.

[2] Zhu S., Wang Y. (2024). A 1.8 V, SNDR 96.3 dB analog front-end circuit design for ECG signal acquisition. Proceedings of IEEE International Conference on Natural Language Processing (ICNLP), Xi’an, China.

[3] Omran A., Aprile A., Moisello A., Tatu V., Calabro R., Malcovati P., Bonizzoni E. (2025). Toward a Generalized Analog Front End for Multiple Biomedical Signals Acquisition: A Review. IEEE Sens. J..

[4] Stanesic A., Klaic L., Cindric D., Cifrek M. (2025). Analog Front End for Capacitive Electrodes in Biomedical Applications. Proceedings of the MIPRO ICT and Electronics Convention, Opatija, Croatia.

[5] Guimaraes V.B., Righi F.Z., Muller C., Aguirre P.C.C., Girardi A.G. (2025). Analog Front-End for ECG Signal Acquisition with Automatic Gain Control. Proceedings of IEEE International Symposium on Instrumentation Systems, Circuits and Transducers (INSCIT), Manaus, Brazil.

[6] Park Y., Mo Y.J., Kim J.H., Cauwenberghs G., Kim S.J. (2025). A 4.6 μW, 133-VPP Common-Mode Interference-Tolerant Biopotential Amplifier for Two-Electrode Recording System in 110-nm CMOS. IEEE J.-Solid-State Circuits.

[7] Hyoung S.G., Koo N. (2026). Common-Mode Interference in Biopotential Amplifiers: Modeling, Analysis, and Design Strategies for Various Recording Setups. IEEE Trans. Biomed. Circuits Syst..

[8] Absi M.A.A., Mohamed A.R. (2024). Design and Characterization of a Configurable Current Conveyor Circuit. Proceedings of the International Conference on Microelectronics (ICM), Doha, Qatar.

[9] Pandiev I.M. (2023). Development of Self-Limiting LC Oscillators Using Cascade Structure of Monolithic CCIIs. Proceedings of the International Conference on Mixed Design of Integrated Circuits and System (MIXDES), Kraków, Poland.

[10] Dash S.K., Bakshi A., Panda J.R., Mishra S.N. (2023). Study of Low Power Techniques in Analog Circuit and Its Application to Design Second Generation Current Conveyors and Voltage Amplifiers. Proceedings of the International Conference on Recent Advances in Electrical, Electronics, Ubiquitous Communication, and Computational Intelligence (RAEEUCCI), Chennai, India.

[11] Sadaghiani S.M., Nguyen H.C., Bhadra S. (2024). Sub-threshold Current Conveyer for Current-Mode Processing Bio-Analog Front Ends. Proceedings of the IEEE International Midwest Symposium on Circuits and Systems (MWSCAS), Springfield, MA, USA.

[12] Silvestre J.L.S. (2021). A 2.24 NEF Current-Balancing Instrumentation Amplifier Using Inverter-Based Transimpedance Stage for ECG Signal Acquisition in 180 nm Technology. Proceedings of Devices for Integrated Circuit (DevIC), Kalyani, India.

[13] Thanapitak S., Pawarangkoon P., Surakampontorn W., Ahmad R., Abdullah Z.R., Manaf A.A., Adirek S., Chanapromma C. (2025). A 38.4 nW, 1.2 V, 250-Hz, 2nd-Order gm-C LPF with Degenerative SCP Transconductors Achieving 800-mVPP Input Range and 82.1-μVrms IRN for ECG Acquisition. IEEE Trans. Circuits Syst. II Express Briefs.

[14] Gu X., Li J., Wu S., Lyu H. (2025). A 1.28-μW Heart-Rate SoC Achieving 99.68% QRS Detection Accuracy for Long-Term Continuous Cardiac Monitoring Applications. IEEE Trans. Very Large Scale Integr. (VLSI) Syst..

[15] Guerm M., Benatti S., Benini L. (2024). A Noncontact ECG Sensing System with a Micropower, Ultrahigh Impedance Front-End, and BLE Connectivity. IEEE Sens. J..

[16] Li D., Hattori R., Matsunuma S. (2024). Capacitive ECG Circuit with Fast Recovery for Continuous Exercise Monitoring. Proceedings of the Annual International Conference of the IEEE Engineering in Medicine and Biology Society (EMBC), Orlando, FL, USA.

[17] Jain S. (2024). Effect of dVout on different Gain for proposed Current Feedback Operational Amplifier. Proceedings of IEEE International Conference for Women in Innovation, Technology & Entrepreneurship (ICWITE), Bangalore, India.

[18] Pantuprecharat P., Sitjongsataporn S., Pawarangkoon P. (2023). 0.5 V DC Suppression, Low Noise, High Impedance CCII-Based Electrocardiogram Amplifier. Int. J. Intell. Eng. Syst..

[19] Sedra A.S., Smith K. (2014). Microelectronic Circuits.

[20] Xing L., Cai Y., Zhang Y., Mottini V., Heller L., Li J. (2025). Skin-conformal electronics for wearable electrogastrography monitoring. IEEE J. Flex. Electron..

[21] Aviles-Espinosa R., Dore H., Rendon-Morales E. (2023). An Experimental Method for Bio-Signal Denoising Using Unconventional Sensors. Sensors.

[22] Rigol DG800 Pro Series. https://www.rigol.com/dam/global/downloads/brochures/en/user-manual/waveform-generators/DG800Pro_UserGuide_EN.pdf.

